# An Autopsy Case of Rapidly Aggravated *Clostridium perfringens* Septicemia with Colorectal Cancer

**DOI:** 10.1155/2022/1071582

**Published:** 2022-09-30

**Authors:** Risako Kohya, Taichi Murai, Yudai Taguchi, Kyohei Sawai, Masaya Takehara, Masahiro Nagahama, Kazufumi Itaya, Yuta Koike, Ayana Endo, Yuji Ono, Atsushi Nagasaka, Shuji Nishikawa, Michio Nakamura

**Affiliations:** ^1^Department of Gastroenterology, Sapporo City General Hospital, 1-1, N-11, W-13, Sapporo 0608604, Japan; ^2^Department of Clinical Laboratory Testing, Sapporo City General Hospital, 1-1, N-11, W-13, Sapporo 0608604, Japan; ^3^Department of Microbiology, Faculty of Pharmaceutical Science, Tokushima Bunri University, Yamashiro-cho, Tokushima 7708514, Japan; ^4^Department of Infectious Diseases, Sapporo City General Hospital, 1-1, N-11, W-13, Sapporo 0608604, Japan

## Abstract

This report presents a case of a 60-year-old man who was diagnosed with ascending colon cancer with metastases of the lymph nodes and multiple liver metastases. Three days before the introduction of the first chemotherapy, he visited our hospital due to high fever. The blood test revealed an increase in the inflammatory response, hepatobiliary enzyme level, lactate dehydrogenase (LDH) level, and renal function deterioration. Contrast-enhanced computed tomography (CT) showed a rapid progression of primary lesion and liver metastatic lesions. Treatment with 5-fluorouracil, leucovorin, and oxaliplatin and cetuximab (FOLFOX/Cmab) was initiated, and the patient was admitted to our hospital after the first day of chemotherapy. At midnight, he had chills, red urine, and rapid hypoxemia. The second blood test showed progression of anemia; increased total bilirubin, aspartate aminotransferase, and LDH levels; and decreased platelet and fibrinogen levels. The serum was red wine in color, indicating marked hemolysis. The respiratory condition rapidly deteriorated, and tracheal intubation was performed and transferred into the intensive care unit. However, blood oxygenation did not increase, and the patient died the next morning, 19 h after admission, despite intensive care. Postmortem CT showed intraperitoneal free air and gas retention in the liver tumor and portal vein system. Pathological autopsy revealed perforation in ascending colon cancer, many Gram-positive rods in the perforation site, dissemination of bacteria throughout the body, and diffuse pulmonary edema. Subsequently, blood cultures reported *Clostridium perfringens* (CP), which is a product of alpha-toxin. CP infection can cause rapid aggravation and sudden death. The physicians should be aware of this highly fatal infection, leading to immediate diagnosis and treatment.

## 1. Introduction

*Clostridium perfringens* (CP) is a causative agent of gas gangrene and food poisoning after trauma. Conversely, cases of nontraumatic CP infection in patients with cancer and diabetes and immunocompromised patients leading to death in a short time have been reported [1].

We report a case of a patient with ascending colon cancer who died in a short time due to severe intravascular hemolysis and multiple organ failure due to CP sepsis.

## 2. Case Report

**Figure 1:**
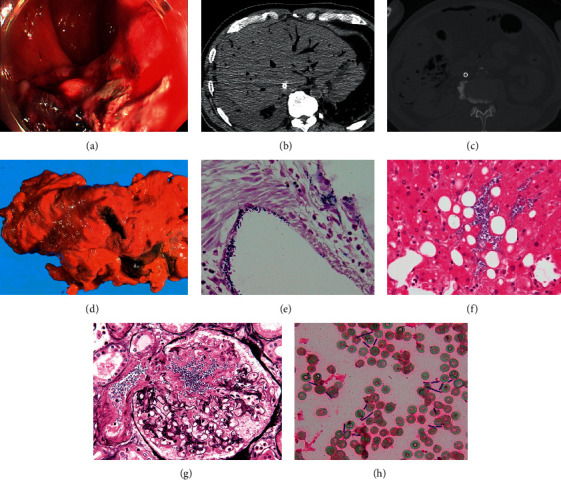
(a) Endoscopy at diagnosis showed a type 2 tumor in the hepatic curvature of the ascending colon. (b)–(c) Autopsy imaging shows gas retention in the tumor and metastasis of the liver and portal vein. There was also free air in the abdominal cavity, suggesting perforation of the gastrointestinal tract. (d) In the pathological autopsy, the foul-smelling gas was removed upon opening the abdomen, and the tumor area in the colon was perforated. (e) Numerous Gram-positive bacilli were found in the perforation of the colon tumor. (f) Numerous Gram-positive rods were found in the liver with gas production. (g) In the kidney, the fungus destroyed the glomerular appendages. (h) Gram staining of the blood confirmed Gram-positive rods.

A 68-year-old man visited our outpatient clinic with a chief complaint of right abdominal pain. He had a history of type 2 diabetes (HbA1c level, 6.1%) and cholecystectomy. Colonoscopy revealed a 3-cm-diameter type 2 colorectal cancer in the liver curvature of the transverse colon (Figure1(a)). Tumor markers were not elevated with carcinoembryonic antigen (CEA) 1.6 ng/ml and carbohydrate antigen 19-9 (CA19-9) 9 U/ml (Table 1). Histopathology revealed differentiated adenocarcinoma. Computed tomography (CT) showed lymph node metastasis and multiple liver metastases, and the patient was diagnosed with transverse colon cancer (T4b, N1, M1, cStage IV, Union for International Cancer Control Staging).

One month after his first visit, three days before the patient's schedule for chemotherapy, he developed high fever of 38.8°C and visited the outpatient clinic.

The physical findings at admission were clear consciousness, performance status of 1, body length of 176.5 cm, body weight of 81.2 kg, body temperature of 37.7°C, blood pressure of 103/62 mmHg, pulse of 105 times/min, and SpO_2_ of 93%–95% (room air). He had mild jaundice in the conjunctiva and no anemia in the eyelids. No abnormal findings in the chest and abdomen were found.

**Figure 2:**
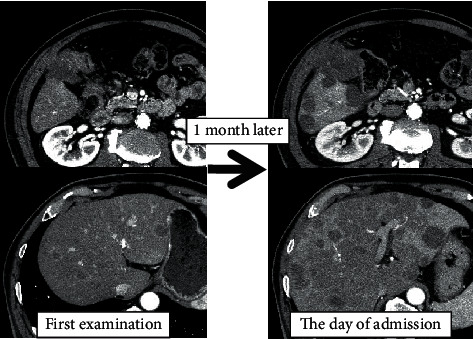
Contrast-enhanced CT on admission shows rapid progression of the primary tumor and liver metastases compared with that in the initial examination 1 month prior.

The laboratory findings showed elevated leukocyte counts of 18700/*μ*L, hemoglobin count of 13.7 g/dL, and C-reactive protein level was 9.4 mg/dL. Liver enzyme levels were also elevated, such as total bilirubin level of 2.6 mg/dL, gamma-glutamyl transpeptidase level of 299 U/L, alkaline phosphatase level of 585 U/L, aspartate aminotransferase (AST) level of 125 U/L, alanine aminotransferase (ALT) level of 133 U/L, and lactate dehydrogenase (LDH) of 1406 U/L. Tumor markers were not elevated with CEA 1.2 ng/ml and CA19-9 15 U/ml (Table 1). Contrast-enhanced CT scan showed rapid progression of the primary tumor and liver metastases compared to the initial visit one month before (Figure 2), and we considered that the fever and changes in the blood data were due to the rapid deterioration of the liver metastases. Therefore, Day 1 treatment of FOLFOX/cetuximab regime, which was already planned, was started immediately. Chemotherapy was started in the outpatient clinic and completed at 17:00 p.m. Then, the patient was admitted to the hospital ward. Vital signs were stable, and he was able to eat dinner thoroughly.

However, the patient suddenly complained of chills at 21:00 p.m., and the body temperature increased to 40°C at 23:00. There were no complaints of abdominal symptoms or other complaints, and symptomatic treatment with antipyretics was administered. The next morning at 4:30 a.m., hematuria was observed, and simultaneously, dyspnea appeared. The blood oxygen level deteriorated rapidly; thus, a second blood test and blood culture were performed. The collected serum was red wine in color, and the blood test results showed that the hemoglobin level had significantly decreased from 13.5 g/dL to 6.5 g/dL compared to that of the previous day when the patient was admitted. Moreover, total bilirubin, AST, and LDH levels were elevated, indicating significant hemolytic anemia (Table 1). Furthermore, there was an elevated inflammatory response and renal dysfunction. At 9:00 a.m., 17 h after admission, the arterial blood gas level deteriorated (PaO_2_ of 50 mmHg), and the patient was transferred to the intensive care unit and on ventilator management. However, oxygenation did not improve after ventilation was started, and the patient developed bradycardia, which led to cardiac arrest. Cardiopulmonary resuscitation was performed, but the patient died at 11:08 a.m., 19 h after admission.

Autopsy CT was performed immediately after death and showed gas retention in the portal vein of the tumor and liver and a small amount of intraabdominal free air (Figures 1(b) and 1(c)).

Pathological autopsy was performed 2 h after death. At the time of laparotomy, gas with a strange odor was removed, and there was perforation in the tumor part of the hepatic curve (Figure 1(d)). Pathological findings showed numerous Gram-positive rods in the perforated part of the tumor (Figure 1(e)). A large number of Gram-positive rods with gas production were also found in the liver (Figure 1(f)). In the kidney, the bacteria destroyed the glomerular capillaries (Figure 1(g)). Gram staining of the blood also showed Gram-positive rods (Figure 1(h)), and blood culture was positive for *C. perfringens*. The production of alpha-toxin in the supernatant of the cultured fungus was demonstrated using western blot analysis. Polymerase chain reaction (PCR) confirmed that the bacterial DNA of this case had the alpha-toxin and enterotoxin genes, and it was an F type bacterium according to the classification of Rood et al. [2].

## 3. Discussion

CP is a spore-forming anaerobic bacterium. This bacterium resides in the soil, sewage, human intestinal tract, and genitourinary system. This bacterium begins to grow rapidly when they meet anaerobic conditions and becomes a causative agent of food poisoning or gas gangrene [3].

CP produces approximately 20 types of toxins, most of which are extracellular and conventionally classified into types A to E according to the pattern of toxins produced and released [4, 5].

Currently, classification into A to F types by toxin gene analysis was recommended. The CP bacterium in this case was considered F type because it had alpha-toxin and CP toxin genes from the PCR analysis [2].

The main toxin that causes gas gangrene in humans is alpha-toxin, and the toxin was confirmed in the CP bacterium culture medium of this case. Alpha-toxin possesses phospholipase C activity, which acts on phosphatidylcholine of the membrane outer leaflet and causes cell lysis.

When CP becomes vegetative from spores, it begins to grow rapidly. Once the bacteria enter the bloodstream and a large amount of alpha-toxin is released into the blood, red blood cells are hemolyzed and destroyed [6].

As risk factors for the sepsis due to *C. perfringens*, trauma, surgery on the large intestine and gallbladder, hemodialysis, diabetes, liver cirrhosis, malignant tumors of the intestinal tract, hematological malignancy, inflammatory bowel disease, neutropenia, and delivery have been reported [7].

CP is present in the normal bacterial flora of the host, and the CP sepsis usually spreads from infections that develop in the body (urinary tract infection, respiratory infection, intraperitoneal infection, cholecystitis, and bloodstream).

Regarding CP infections associated with malignant tumors, colorectal cancer is the most common lesion, followed by pancreatic cancer and gastric cancer [8].

This case was a very instructive case and worth reporting. Because the lethal sepsis simultaneously developed when initiation of chemotherapy was necessary and urgent.

Rapid worsening of the colon cancer and exacerbated hepatic dysfunction led the diagnosis that the origin of fever was more likely caused by tumor progression rather than by infection. This case taught us that it is essential to keep in mind that serious infection such as sepsis caused by this bacterium may be lurking behind rapidly progressing gastrointestinal cancer with unexpected fever.

This case was considered at high risk due to immunosuppression because the patient had developed colorectal cancer with multiple liver metastases and poorly controlled diabetes mellitus. In poorly differentiated and highly proliferated colorectal cancer, as in the present case, the anaerobic state continues, and it is highly likely that CP bacteria multiply rapidly, disseminate to the liver via the portal vein, and spread throughout the body in the bloodstream.

This bacterial sepsis has an extremely poor prognosis, and the mortality rate of 50 cases with intravascular hemolysis is 74%, and the average time from visit to death is only 9.7 h [1]. The patient died 19 h after admission. Although the patient presented high fever at the time of admission, the cause of fever was thought to be the rapid progression of colorectal cancer, and chemotherapy was started on the same day. However, this is evident that the bloodstream infection had already occurred and fever was a symptom of CP infection. In this case, involvement of chemotherapy in the sepsis and intestinal perforation is considered small, because it was extremely early for the antitumor effect of chemotherapy and development of immunosuppression. To improve the survival rate of the CP sepsis, it is necessary to diagnose and initiate treatment as early as possible. However, identification of the causative bacteria requires bacterial culture and genetic tests, and it take days for the results to be obtained. Therefore, even if treatment is initiated as soon as the bacteria have been identified, there is a high possibility that the patients may not survive.

CP infections often cause gas gangrene, and the clinical image is extremely similar to that of invasive streptococcal group A infections, which cause necrosis of the skin and soft tissues, but blood smears and Gram stains of pus are useful to distinguish them. CP is classified as a Gram-positive bacillus and can be easily distinguished from the Gram-positive streptococcus. Furthermore, *Klebsiella pneumoniae* and *Escherichia coli*, which cause gas-producing abscesses, are Gram-negative bacilli. This case, although it was after death, confirmed a positive bacillus by Gram stain of peripheral blood smear. Gram stain of both peripheral blood and abscess discharge can be useful in obtaining a diagnosis in a short time [9].

For the treatment of CP infection, it is essential to start intravenous antibiotic administration as early as possible and surgical debridement if possible [10]. A broad-spectrum antibiotic that not only covers CP bacteria but is also effective for *Staphylococcus aureus*, *Streptococcus* spp., *Enterobacteriaceae*, *Pseudomonas aeruginosa*, and *Bacteroides* spp., regardless whether aerobic or anaerobic, must be selected. Clindamycin suppresses the production of toxins by CP and group A streptococcus, and the combination of penicillin and clindamycin is one of the recommended therapies [11]. Intensive and multidisciplinary care strategies, such as plasma exchange, appropriate fluid therapy, blood transfusion, nutritional control, blood pressure control, and dialysis therapy, are highly recommended to increase the survival rate in CP infection [12]. Kubo reported that early initiation of the endotoxin adsorption therapy was effective for the CP-induced sepsis [13]. As described before, if Gram-positive stain of peripheral blood smear had been examined at this patient's hospitalization, we might have been confirmed a positive bacillus rather quickly. Then, early initiation of multidisciplinary treatments might have successfully saved the patient.

As risk factors for infectious diseases of this bacterium, old age, malignant tumor, diabetes, peptic ulcer, and immunodeficiency, have been reported. If high risk patients have fever, gastrointestinal symptoms, hemolytic anemia, or impaired consciousness, this infection should be in the list of differential diagnosis. Once confirmed, antibiotic treatment and multidisciplinary treatment, including drainage and debridement, if necessary, should be initiated as early as possible.

## 4. Conclusion

In this case, a patient with risk factors, such as old age and diabetes mellitus, developed a rapidly growing poorly differentiated colorectal tumor, which led to extensive spread of CP bacteria under anaerobic conditions. Subsequently, CP bacteremia resulted in severe hemolysis and multiple organ failure, and the patient died within a short period. If patients with similar backgrounds have fever, inflammatory findings, or hemolytic anemia, CP infection must be distinguished by careful examination and necessary tests.

## Figures and Tables

**Table 1:** Laboratory findings.

	The day of admission (day 1)	The day of emergency (day 2)
WBC (/*μ*l)	18.7 × 10^3^	19.9 × 10^3^
Hgb (g/dl)	13.7	6.5
PLT (/*μ*l)	215 × 10^3^	32 × 10^3^
T-Bil (mg/dl)	2.6	15.8
*γ*-GT (U/l)	299	241
ALP (U/l)	585	784
AST (U/l)	125	1037
ALT (U/l)	133	170
LD (U/l)	1406	10602
ALB (g/dl)	3.7	3.3
TP (g/dl)	7.2	4.7
UN (mg/dl)	19.2	49.8
Cr (mg/dl)	1.2	1.9
Na (mEq/l)	135	132
K (mEq/l)	4.3	5.8
Cl (mEq/l)	98	95
Ca (mg/dl)	8.8	6.8
iP (mg/dl)	3.4	4.3
UA (mg/dl)	5.1	9.8
CRP (mg/dl)	9.4	14.11
PT (%)		21
PT-INR		2.55
APTT (sec)		81
